# Effects of orlistat combined with enzalutamide and castration through inhibition of fatty acid synthase in a PC3 tumor-bearing mouse model

**DOI:** 10.1042/BSR20204203

**Published:** 2021-05-27

**Authors:** Yeu-Sheng Tyan, Yen-Po Lee, Hui-Yen Chuang, Wei-Hsun Wang, Jeng-Jong Hwang

**Affiliations:** 1 Department of Medical Imaging, Chung Shan Medical University Hospital, Taichung, Taiwan, ROC; 2 Department of Medical Imaging and Radiological Sciences, Chung Shan Medical University, Taichung, Taiwan, ROC; 3 Department of Biomedical Imaging and Radiological Sciences, National Yang Ming Chiao Tung University, Taipei, Taiwan, ROC; 4 Department of Orthopedic Surgery, Changhua Christian Hospital, Changhua, Taiwan, ROC; 5 Department of Medical Imaging and Radiology, Shu-Zen Junior College of Medicine and Management, Kaohsiung, Taiwan, ROC

**Keywords:** castration therapy, enzalutamide, fatty acid synthase, NF-kB, orlistat, prostate cancer

## Abstract

Androgen deprivation therapy (ADT) is one of the typical treatments used for patients with prostate cancer (PCa). ADT, however, may fail when PCa develops castration-resistance. Fatty acid synthase (FASN), a critical enzyme involved in fatty acid synthesis, is found to be up-regulated in PCa. Since enzalutamide and ADT are frequently used for the treatment of PCa, the present study aimed to unravel the underlying mechanism of combination of orlistat, an FASN inhibitor, and enzalutamide using PC3 cell line; and orlistat and castration in PC3 tumor-bearing animal model. Cytotoxicity was determined by AlamarBlue assay. Drug effects on the cell cycle and protein expressions were assayed by the flow cytometry and Western blot. Electromobility shift assay was used to evaluate the NF-κB activity. The tumor growth delay, expressions of the signaling-related proteins, and histopathology post treatments of orlistat and castration were evaluated in PC3 tumor-bearing mouse model. The results showed that orlistat arrested the PC3 cells at the G_1_ phase of the cell cycle and enhanced the cytotoxic effects of enzalutamide synergistically. Pretreatment with orlistat combined with castration inhibited the tumor growth significantly compared with those of castration and orlistat treatments alone in PC3 tumor-bearing mice. Combination treatment reduced both FASN and NF-κB activities and their downstream effector proteins. The present study demonstrated the synergistic effects of orlistat combined with enzalutamide *in vitro* and castration *in viv*o on human PCa.

## Introduction

Prostate cancer (PCa) is the second most frequent cancer and the fifth leading cause of cancer-associated mortality in men [1]. Radiation therapy (RT) is the typical treatment used for localized PCa [2]. However, RT has a 20–30% failure rate and some cases even develop into metastasis post-RT [3]. For more advanced and metastatic PCa, the gold standard strategy is androgen deprivation therapy (ADT) [4], which is initially highly effective at reducing tumor size, lowering prostate-specific antigen (PSA) levels and extending survival time [5]. However, persisting ADT often provides selective pressure on cancer cells and converts them to become more aggressive and treatment-resistant, leading to the development of androgen-independent PCa (AIPC) or castration-resistant PCa (CRPC) [6,7]. These alterations would increase the mortality rates due to the lack of effective alternative targeted therapy [8]. Bypass pathways, which play important roles in AIPC, are closely associated with the activation of various oncogenes that are involved in promoting proliferation and escaping apoptosis [9]. Signals from activated receptor tyrosine kinases (RTKs), Ras-Raf-MEK-ERK1/2 and PI3K/AKT machineries in turn can activate several transcription factors, such as NF-kB and its downstream effector proteins including MMP-9, cyclooxygenase-2, cyclin D1, EMT, VEGF and Bcl-2. The aforementioned proteins were reported to promote tumor progression and cause resistance for ADT and RT in PCa [10–15]. Recently, a combination treatment of enzalutamide and ADT for metastatic hormone-sensitive PCa (mHSPC) in phase III clinical trial has been reported. These reports concluded that enzalutamide offers a benefit in treatment post-docetaxel in mHSPC [16,17].

A hallmark of PCa progression is the dysregulation of lipid metabolism via overexpression of fatty acid synthase (FASN), the key enzyme in *de novo* fatty acid synthesis [18]. Numerous malignancies, including prostate, colon, breast and lung carcinomas, were found to express higher levels of FASN compared with their non-tumor counterparts, and overexpressed FASN was associated with poor prognosis [19–22]. In PCa cells, FASN could be regulated by androgen-dependent and -independent machineries via activating the androgen receptor (AR) and Ras-related and PI3K-related pathways [23,24]. FASN overexpression could also convert PCa into CRPC [24]. Long-chain fatty acids are the components of phospholipids, which are important for cell membrane integrity, especially the lipid rafts [25,26]. A number of receptors are located in the lipid rafts and trigger various cascade signal transduction closely associated with the proliferation, survival, angiogenesis and metastases of cancer cells. Disruption of the lipid rafts and modification of the cell membranes by uptake of more polyunsaturated fatty acids was proven to be able to reduce tumor growth both *in vitro* and *in vivo* [26–28]. In addition, long-chain fatty acids are critical for the maturation and modification of the proteins, especially the localization and stimulation of receptors in charge of major oncogenic signaling pathways [29]. Therefore, FASN inhibition may affect lipid-associated intracellular signal transduction pathways such as androgen–androgen receptor, Ras-Raf-MEK-ERK1/2 and PI3K/AKT signaling pathways [30].

Orlistat could inhibit the synthesis of FASN via binding to the thioesterase domain of FASN [31]. Orlistat was demonstrated to have antitumor effect other than anti-obesity [32]. Furthermore, PCa treated with orlistat could be arrested at the G_1_ phase, decreased FASN activity, suppressed metastasis, angiogenesis and proliferation, and promoted the cancer cells to undergo apoptosis [33–36]. Our pervious study showed that orlistat could enhance RT outcomes by inhibiting FASN and NF-κB activities in androgen-dependent and -independent PCas [37]. The present study evaluated the combination effects of orlistat with enzalutamide and castration in PC3 cells and tumor-bearing animal model, respectively. The results showed that orlistat could enhance the outcomes significantly through inhibition of FASN and NF-κB activities and phosphorylations of AKT and ERK pathways.

## Materials and methods

### Cell culture

The human PCa PC3 cell line was purchased from the American Type Culture Collection. PC3 cells were maintained in F-12K medium (cat. no. 45000-35; Corning Inc.) supplemented with 10% fetal bovine serum (cat. no. SH30080.03; HyClone; Cytiva) and 1% penicillin/streptomycin (cat. no. 15140-122; Gibco; Thermo Fisher Scientific, Inc.). All cells were maintained at 37°C in a humidified incubator containing 5% CO_2_.

### Preparation of orlistat and enzalutamide solution

For *in vitro* experiments, orlistat was dissolved in absolute ethanol (cat. no. 15055, Electron Microscopy Sciences). Enzalutamide was purchased from Selleck Chemicals (cat. no. S1250) and dissolved in 100% DMSO (cat. no. 410301; BioLife Solutions). The final concentration of ethanol or DMSO was 1% when applied to these drugs in cytotoxicity assays, Western blotting and electrophoretic mobility shift assays (EMSAs). For *in vivo* studies, orlistat was extracted from Zerocal capsules (cat. no. 048292 G-9297; Anxo, Taipei, Taiwan) according to a study by Kridel et al. [32]. A capsule of orlistat (120 mg) was dissolved in 0.33 ml absolute ethanol (cat. no. 15055; Electron Microscopy Sciences) and 0.67 ml PEG400 (cat. no. 25322-68-3; Sigma–Aldrich; Merck KGaA) for 30 min, vortexed every 10 min and centrifuged at 13000 rpm for 10 min to remove the insoluble pellets. The supernatant was stored at −80°C. The mice were treated with 240 mg/kg body weight/day via intraperitoneal (*i.p.*) injection.

### Cytotoxicity of orlistat and enzalutamide on PC3 cells

A total of 5 × 10^3^ PC3 cells were seeded into a 96-well plate. On the following day, the cells were treated with serial concentrations (0–250 μM) of orlistat, enzalutamide and orlistat with enzalutamide for 48 h. The cell viability of PC3 cells was determined using an AlamarBlue assay. Briefly, 10 μl (10% of culture medium) of AlamarBlue reagent (cat. no. BUF012A; Bio-Rad Laboratories, Inc.) was added and incubated with cells for 4 h at 37°C. The absorbance at wavelengths of 570 and 600 nm was measured using an ELISA reader (Tecan 200/200PRO; Tecan Group, Ltd.). The cell viability was calculated by the equation provided by the manufacturer: (117216 × A1-80586 × A2)/(155677 × N2-14652 × N1) × 100; where A1: absorbance of tested well at 570 nm; A2: absorbance of test well at 600 nm; N1: absorbance of negative control well (medium plus AlamarBlue but no cells) at 570 nm; N2: absorbance of negative control well at 600 nm. The combination index (CI) was calculated as follows. For the IC_50_ dose, CI = (D)_C_/(D_X_)_1_ + (D)_C_/(D_X_)_2_; where (Dx)_1_ is for (D)_1_ ‘alone’ that inhibits a system x%, and (Dx)_2_ is for (D)_2_ ‘alone’ that inhibits a system x% whereas in the numerator, (D)_C_ is ‘in combination’ also inhibits x%. (D_1_) refers to drug A, (D_2_) refers to drug B, (D_c_) refers to the combination of drugs A and B. The CI value quantitatively defines the synergism (CI < 1), addition (CI = 1) and antagonism (CI > 1) [38].

### Flow cytometry analysis of cell cycle status

A total of 1.5 × 10^6^ PC3 cells were cultured on 10-cm-diameter dishes. On the following day, cells were treated with serial concentrations of orlistat, enzalutamide and orlistat combined with enzalutamide for 48 h. Cells were then trypsinized and resuspended in ice-cold PBS and fixed with ice-cold 70% ethanol in PBS at −20°C overnight. After PBS washing, cells were stained with 1 ml propidium iodide (PI) solution (cat. no. P4170; Sigma–Aldrich; Merck KGaA) [20 μg/ml PI + 200 μg/ml RNaseA (cat. no. E866; Amresco LLC) + 0.1% (v/v) Triton X-100 in PBS] in the dark for at least 30 min at room temperature and then analyzed with an FACScan flow cytometer (CytoFLEX; Beckman Coulter, Inc.). Data were analyzed using CytExpert Software v2.3 (CytoFLEX; Beckman Coulter, Inc.).

### Western blotting

A total of 1.5 × 10^6^ PC3 cells were cultured on 10-cm-diameter dishes. On the following day, cells were treated with serial concentrations of orlistat, enzalutamide and orlistat combined with enzalutamide for 48 h. Cell and tumor lysates were prepared using lysis buffer (50 mM Tris-HCl pH 8.0; 120 mM NaCl; 0.5% NP-40) and tissue protein extraction reagent (cat. no. 78510; TPER; Thermo Fisher Scientific, Inc.), respectively. Equal amounts of lysates were separated by SDS/PAGE. Separated proteins were transferred to PVDF membranes (cat. no. 79548A; Pall Life Sciences), then blocked with 5% non-fat milk or 5% BSA in TBS-Tween-20 (TBS-T). The membranes were then incubated with primary antibodies against FASN (cat. no. 3180), NF-κB p65 (cat. no. 8242), phosphorylated-AKT (cat. no. 4060), AKT (cat. no. 4691; Cell Signaling Technology, Inc.), phosphorylated-p44/42 MAPK (ERK1/2) (cat. no. 4370; Cell Signaling Technology, Inc.), p44/p42 MAPK (Erk1/2) (cat. no. 4695; Cell Signaling Technology, Inc.), Bcl-2 (cat. no. 15071; Cell Signaling Technology, Inc.), cleaved caspase-3 (cat. no. 9664; Cell Signaling Technology, Inc.), MMP-9 (cat. no. AB19016; EMD Millipore), VEGF (cat. no. ABS82; EMD Millipore), cyclin D1 (cat. no. GTX108624; GeneTex, Inc.) and β-actin (cat. no. GTX109639; GeneTex, Inc.) at 4°C overnight. After washing in TBS-T, membranes were incubated with horseradish peroxidase-conjugated secondary antibodies including rabbit IgG (cat. no. GTX213110-01; GeneTex, Inc.) and mouse IgG antibody (cat. no. GTX213111-01; GeneTex, Inc.) at room temperature for 1 h. Protein bands were visualized using a luminescence imaging system (LAS-4000; (Fujifilm) with an enhanced chemiluminescence detection system (cat. no. WBKLS0500; EMD Millipore). Band intensity was quantified using ImageJ 1.50i (National Institutes of Health) and β-actin was used as an internal control.

### Electromobility shift assay

A total of 1.5 × 10^6^ PC3 cells were cultured on 10-cm-diameter dishes. On the following day, cells were treated with serial concentrations of orlistat, enzalutamide and orlistat combined with enzalutamide for 48 h. Cell and tumor lysates were prepared using lysis buffer (50 mM Tris-HCl pH 8.0; 120 mM NaCl; 0.5% NP-40) and tissue protein extraction reagent (cat. no. 78510; TPER; Thermo Fisher Scientific, Inc.), respectively. Nuclear protein was extracted using the Nuclear Extraction kit (cat. no. 2900; EMD Millipore) according to the manufacturer’s instructions. The following DNA sequences were synthesized for assessing NF-κB activity using EMSA: sense, AGTTGAGGGGACTTTCCCAGGC (oligo ID, 1914063653; Protech Technology, Taipei, Taiwan) and antisense, GCCTGGGAAAGTCCCCTCAAC (oligo ID, 1914063652; Protech Technology, Taipei, Taiwan). NF-κB /DNA binding activity was measured using the LightShift Chemiluminescent EMSA kit (cat. no. 20148; Thermo Fisher Scientific, Inc.) according to the manufacturer’s instructions. Nuclear extracts were incubated with a biotin-labeled DNA probe for 20 min at room temperature. The DNA–protein complexes were separated from free oligonucleotides on 5% polyacrylamide gels, transferred to nylon membranes (cat. no. 75824C; Pall Life Sciences) and cross-linked by UV light. The membranes were incubated with streptavidin–horseradish peroxidase (cat. no. 89880D; Thermo Fisher Scientific, Inc.), and detected by enhanced chemiluminescence (cat. no. 89880E; Thermo Fisher Scientific, Inc.). Band intensities were quantified using ImageJ 1.50i (National Institutes of Health).

### Animals

Six-week-old male nude mice were purchased from the National Laboratory Animal Center, Taiwan. Experiments were conducted in the Animal Center of National Yang-Ming University in accordance with the National Research Council Publication Guide for the Care and Use of Laboratory Animals, and were approved by the Institutional Animal Care and Use Committee of National Yang-Ming University with approval No. IACUC1031256. Nude mice were reared in individually ventilated cages (IVCs) with the temperature between 18 and 26°C, moisture between 30 and 70%, and the noise under 65 dB. The food, water and the bedding were replaced twice a week.

### Orlistat combined with castration in PC3 mouse xenograft

A total of 2 × 10^6^ PC3 cells mixed with 50% Matrigel (cat. no. 354234; BD Biosciences) were inoculated into the right flank of 6-week-old mice to generate a mouse xenograft model. The tumor volume was measured with a digital caliper. When the average tumor size reached ∼100 mm^3^, mice were randomly divided into four groups: control, castration alone, orlistat alone and orlistat combined with castration. For castration, surgery was performed on mice when the tumor size reached 200 mm^3^. Briefly, mice were anesthetized with 2.5% isoflurane in an induction chamber and placed on a stage covered with sterile sheets. A 1-cm incision was made with a scalpel above the scrotum, the testes were pushed out and cut, and the incisions were sutured with non-absorbable suture. For the orlistat combined with castration group, orlistat were administered 3 days prior to surgery. Mice were treated with orlistat daily up to 8 weeks.

### Tissue preparation for histopathology

At the end of the experiments, mice were killed with carbon dioxide asphyxiation, then the tumors, livers and kidneys were removed and fixed in cold 4% paraformaldehyde, embedded in paraffin, sectioned into 5-µm-thick sections and stained with Hematoxylin and Eosin. Slides were observed and photos were taken using a virtual slide system (Olympus BX61; Olympus Corporation).

### Immunohistochemical staining

The paraffin-embedded tissue sections were maintained at 60°C for 1 h and then de-paraffinized in xylene (Sigma–Aldrich; Merck KGaA). The tissue sections were rehydrated in graded ethanol from 95 to 75% and PBS with 0.05% Tween-20 (PBS-T). For antigen retrieval, tissue slides were boiled in 10 mM citric acid buffer with 0.05% Tween-20 (pH 6.0) for 3 min at 121°C using a pressure cooker. After air cooling, the tissue sections were incubated with peroxidase blocking reagent (RTU, EnVision™+Dual Link System-HRP (DAB+); K4065; Dako; Agilent Technologies, Inc.) for 5 min, and then blocked with goat serum for an additional 30 min. Subsequently, the tissue sections were incubated with anti-FASN (cat. no. 3180), NF-κB p65 (cat. no. 8242), p-AKT (cat. no. 4060) and phosphor-p44/42 MAPK (ERK1/2) (cat. no. 4370) antibodies (Cell Signaling Technology, Inc.) at 4°C overnight followed by incubation with horseradish peroxidase-conjugated secondary antibodies. The sections were rinsed with PBS-T, developed in 3′,3′-diaminobenzidine (DAB) substrate chromogen (EnVision™+Dual Link System-HRP(DAB+); K4065; Dako; Agilent Technologies, Inc.), and then counterstained with Mayer’s Hematoxylin (ScyTek Laboratories). All sections were scanned on an Aperio Digital Pathology Slide Scanner (Leica Biosystems).

### Biochemical analysis

At the end of the experiments (after 8 weeks of drug administration), the mice were killed. Serum was collected and analyzed for levels of alkaline phosphatase (ALP), albumin (ALB), alanine aminotransferase (ALT), aspartate aminotransferase (AST), blood urea nitrogen (BUN) and creatinine (CRE). These measurements were performed on the Fuji Dri-Chem 4000i system (Fujifilm). Reference range of biochemical data was obtained from Charles River Laboratories, Inc.

### MicroPET imaging

^18^F-FDG/microPET imaging provides a non-invasive way to detect tumor progression and analyze the metabolic function of cancers. MicroPET studies were performed using the R4 system (R4; Concorde Microsystems). Briefly, mice were injected with 19.6–20.1 MBq ^18^F-FDG intravenously. Thirty minutes later, mice were anesthetized using 2.5% isoflurane, and imaging data were collected for 30 min with the microPET scanner. Regions of interest (ROIs) were drawn over the tumor area using the standardized uptake value (SUV) and calculated by PMOD 4.0 (PMOD Technologies LLC).

### Statistical analysis

All data were presented as the mean ± SD. Data were analyzed using Student’s *t* test and two-way ANOVA on GraphPad Prism 6 (GraphPad Software, Inc., U.S.A.). Tukey’s multiple comparisons test was used for post-hoc analysis following ANOVA. The significance was defined as *P*<0.05 or less.

## Results

### Orlistat synergistically enhances the cytotoxic effects of enzalutamide on PC3 cells

**Figure 1:**
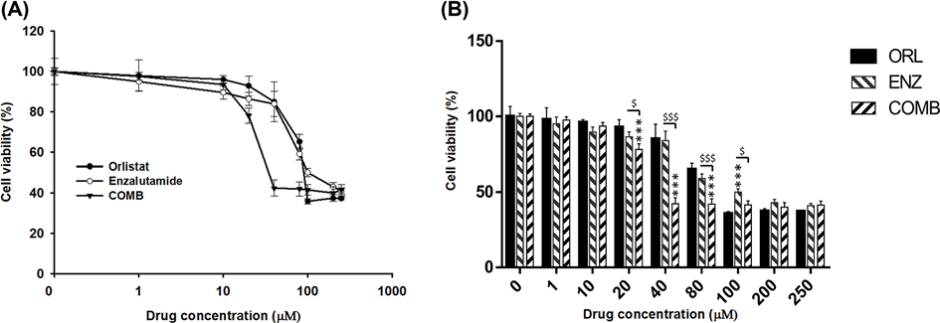
Orlistat increases the therapeutic effects of enzalutamide on PC3 cells (**A**) A total of 5 × 10^3^ PC3 cells/well were seeded into a 96-well plate. The next day, cells were treated with different doses of orlistat alone, enzalutamide alone and a combination of orlistat plus enzalutamide for 48 h. The cell viabilities were evaluated with an AlamarBlue assay. The calculated IC_50_ of orlistat alone, enzalutamide alone and combination treatment (COMB) were 80, 100 and 35 µM, respectively, in PC3 cells. (**B**) Quantification of (A). All experiments were repeated at least three times. ***P<0.001 vs. orlistat alone. ^$^*P*<0.05 and ^$$$^*P*<0.001 vs. combination and enzalutamide alone.

AlamarBlue assay was used to determine the cytotoxicity of orlistat and enzalutamide in PC3 cells. Figure 1 showed that the IC_50_ of orlistat alone, enzalutamide alone and the combination treatment were 80, 100 and 35 µM in PC3 cells, respectively. The CI calculated according to Chou and Talalay [38] was 0.79 (<1), suggesting that the cytotoxic effects of combination treatment was synergistic. These results were also tabulated in Table 1.

**Table 1:** The IC_50_ and CI of different treatments in PC3 cells

Treatments	IC_50_ (μM)
Orlistat group	80
Enzalutamide group	100
Combination group	35
CI	0.79

The CI of combination treatment in PC3 cells was calculated according to Chou and Talalay [38] as CI = (D)_C_/(D_X_)_1_ + (D)_C_/(D_X_)_2_ (36). (D)_C_, (D_X)1_ and (Dx)_2_ stand for IC_50_ of the combination treatment, orlistat alone and enzalutamide alone, respectively. It is synergistic when CI < 1, additive when CI = 1, and antagonistic when CI > 1. Here the CI was calculated as (35/80) + (35/100) = 0.79 (<1), and was identified as synergistic.

### Orlistat, enzalutamide and combination treatment arrests the cell cycle at the G_1_ phase and decreases S and G_2_/M phase fractions in PC3 cells

**Figure 2:**
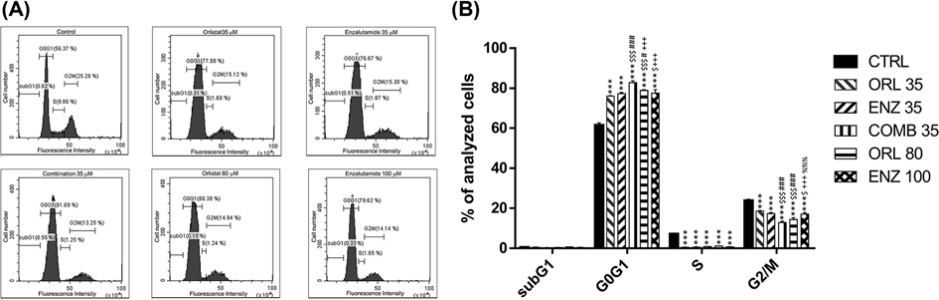
Cell cycle distribution A total of 1 × 10^6^ PC3 cells were cultured in 10-cm dishes 1 day before treatment with different doses of orlistat alone (35 and 80 µM), enzalutamide alone (35 and 100 µM) and the combination of orlistat plus enzalutamide (35 µM orlistat + 35 µM enzalutamide, designated as COMB 35) for 48 h. (**A**) The PC3 cells were arrested at the G_0_/G_1_ phase and the S phase proportion was significantly reduced in all treatments, with the combination treatment showing the most significant effects. (**B**) Quantification of (A). ***P<0.001 vs. control. ^$^*P*<0.05 and ^$$$^*P*<0.001 vs. orlistat alone (35 µM). ^#^*P*<0.05 and ^###^*P*<0.001 vs. enzalutamide alone (35 µM) group. ^+++^*P*<0.001 vs. combination (35 µM). ^%%%^*P*<0.001 vs, orlistat alone (80 µM). All experiments were repeated at least three times.

Flow cytometry was used to analyze how orlistat and enzalutamide affects the cell cycle in PC3 cells. Orlistat alone, enzalutamide alone and the combination treatment all resulted in significant G_1_-phase accumulations, S-phase reduction and decreased G_2_/M-phase in PC3 cells (Figure 2A). The DNA histograms demonstrated that both orlistat alone and enzalutamide alone could arrest the cell cycle at the G_1_ phase and reduce the proportions of S and G_2_/M phases. The changes in cell cycle distribution occurred in a dose-dependent manner for all treatments. Furthermore, the combination treatment group showed the most obvious G_1_-phase accumulations and decreased G_2_/M-phase (Figure 2B).

### Orlistat, enzalutamide and combination treatment reduce the expression of tumor progression-related protein in PC3 cells

**Figure 3:**
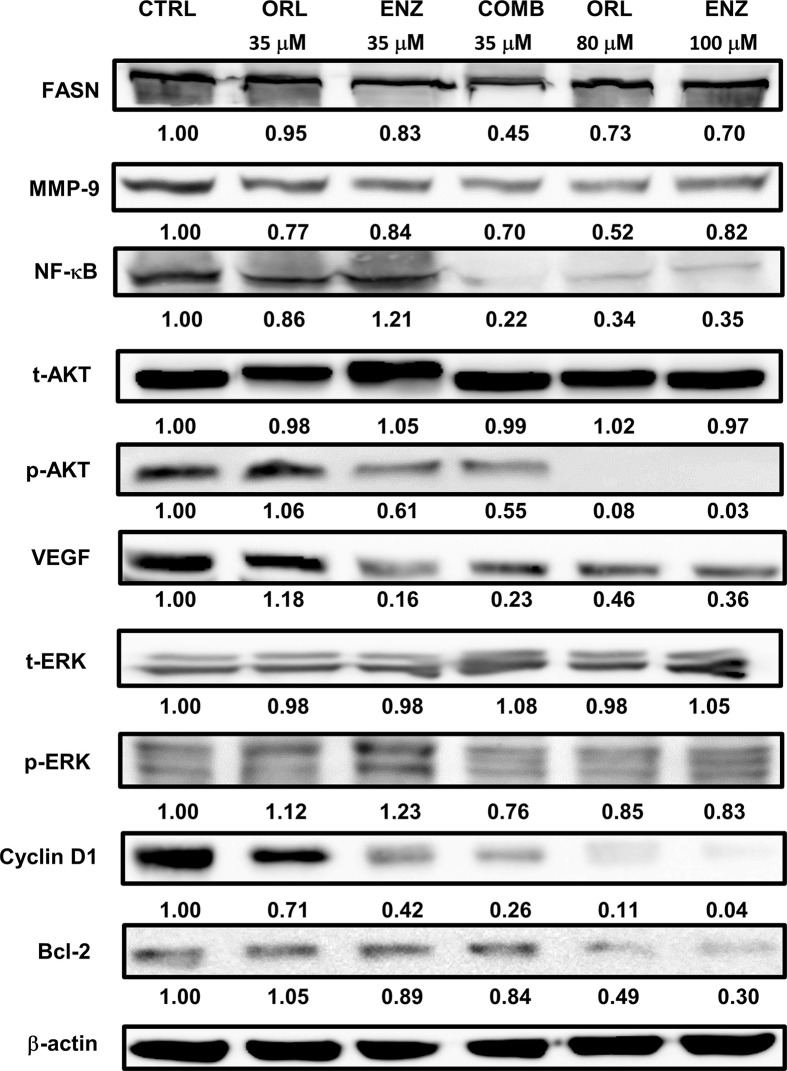
Combination of orlistat with enzalutamide enhances the reduction of related proteins by inhibiting the phosphorylation of AKT and ERK proteins The cell culture conditions and drug treatments were as described in the legend of Figure 2. The results showed that orlistat, enzalutamide and combination treatments all reduced the expression of FASN, MMP-9, NF-κB, p-AKT, VEGF, p-ERK, cyclin D1 and Bcl-2 in PC3 cells. Both orlistat and enzalutamide suppressed protein expression in a dose-dependent manner. All experiments were repeated at least three times for the calculation of alteration in fold, while only one repeat was randomly selected for presentation. Abbreviation: p, phosphorylated.

Figure 3 showed that orlistat alone, enzalutamide alone and the combination treatment groups (35 µM orlistat + 35 µM enzalutamide) reduced the expression of FASN, MMP-9, NF-κB p65 (except 35 µM enzalutamide), VEGF (except 35 µM orlistat), cyclin D1 and Bcl-2 (except 35 µM orlistat) via inhibiting the phosphorylation of AKT and ERK (except 35 µM orlistat and enzalutamide, respectively in PC3 cells. Both orlistat and enzalutamide suppressed the expression of these proteins in a dose-dependent manner. Although the doses were relatively low, combination treatment showed similar results as those observed in groups treated with high concentrations of orlistat or enzalutamide. The original blot images of Figure 3 were shown in the Supplementary Figures S1 and S3.

### Orlistat, enzalutamide and combination treatment decrease NF-κB activity in PC3 cells as detected by EMSA

**Figure 4:**
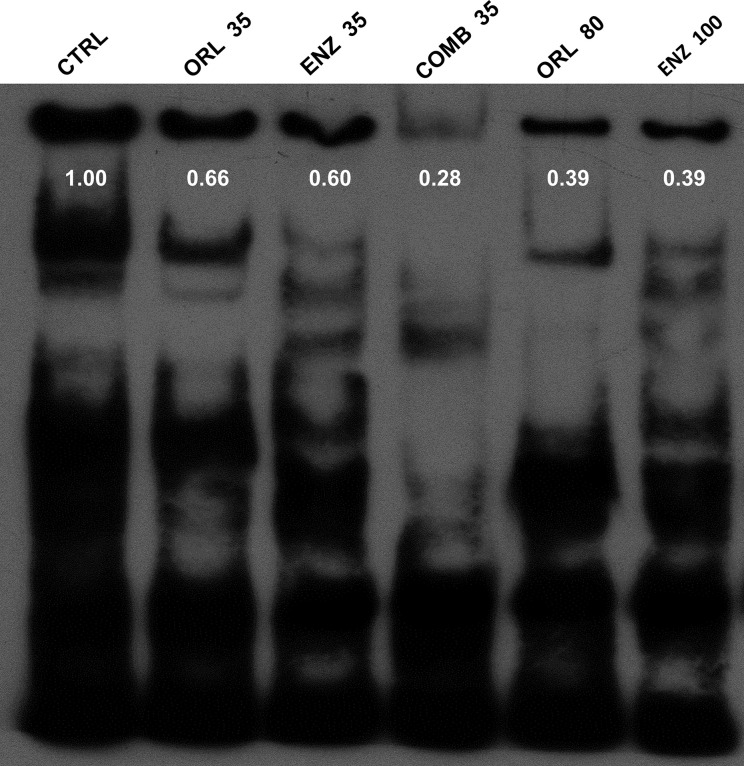
Orlistat, enzalutamide and the combination of both decrease NF-κB activity determined with EMSA The cell culture conditions and drug treatments were as described in the legend of Figure 2. Both orlistat alone (ORL 35 and 80 µM) and enzalutamide alone (ENZ 35 and 100 µM) suppressed the activity of NF-κB in a dose-dependent manner. Combination treatment showed the most significant inhibition of NF-κB activity with relatively low doses. All experiments were repeated at least three times, while only one repeat was randomly selected for presentation.

p-AKT has been known to be the upstream signaling molecule and the proteins detected in Figure 3, in which the transcription factor NF-κB plays an important role. EMSA was performed to determine the activity of NF-κB following these treatments. Figure 4 showed that NF-κB activity was decreased in PC3 cells by orlistat and enzalutamide alone or in combination in a dose-dependent manner. Although the doses were relatively low, the combination treatment showed similar results as those treated with high concentrations of orlistat alone or enzalutamide alone. The results suggested that the combination of orlistat plus enzalutamide treatment could decrease the NF-κB activity at relatively low doses, which is informative for clinical practice.

### Pretreatment with orlistat combined with castration inhibits tumor growth in PC3 tumor-bearing mice

**Figure 5:**
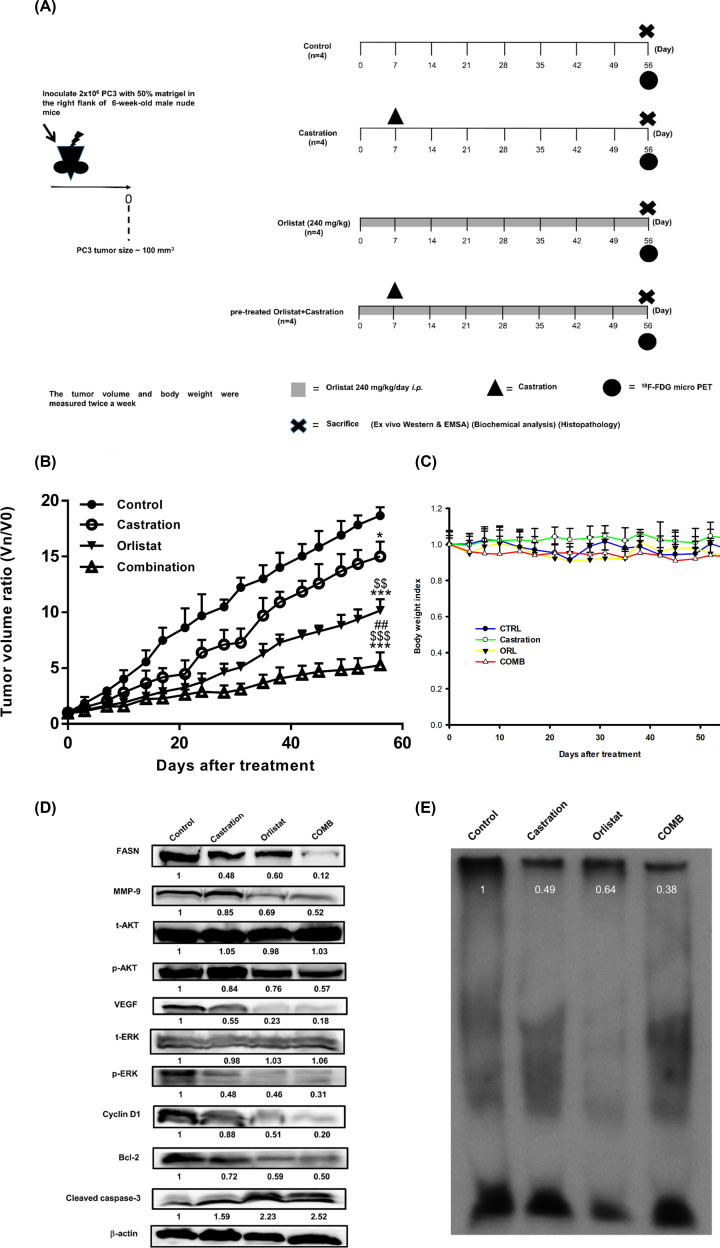
Combination of orlistat with castration improves the therapeutic efficacy in PC3 tumor-bearing mice (**A**) The experimental protocol of the *in vivo* study. (**B**) Tumor growth curve post-treatment assessed via digital caliper measurement. The tumor volume was significantly suppressed by the pretreatment of orlistat plus castration. (**C**) Body weight index demonstrated that the body weight change was within 20% of the original, and no significant difference was found among the groups. (**D**) *Ex vivo* Western blotting results showed that all treatments decreased the expression of FASN, MMP-9, NF-κB, p-Akt, VEGF, p-ERK, cyclin D1 and Bcl-2, while cleaved caspase-3 was increased. The effects of the combination group were significantly better compared with any single treatment. (**E**) The EMSA showed that NF-κB activity was suppressed in all three treatment groups, with combination being the most effective. *P<0.05 and ***P<0.001, all treatments vs. control; ^$$^*P*<0.01, and ^$$$^*P*<0.001, orlistat and combination vs. castration; ^##^*P*<0.01, orlistat vs. combination. *n*=4 mice per group. All experiments were repeated at least twice. Abbreviation: p, phosphorylated.

Based on the *in vitro* results, the present study further examined the therapeutic efficacy of combination treatment in a PC3 xenograft mouse model. The *in vivo* experiments and designed treatment schedules are shown in Figure 5A. The tumor growth curve (Figure 5B) showed that combination of orlistat with castration resulted in the most significant tumor growth inhibition among all groups. On day 56, the average tumor volume ratio of the control, castration, orlistat and combination groups were 1850, 1450, 1000 and 505 mm^3^, respectively. Significant differences in tumor volume between orlistat and combination groups emerged from day 49 (*P*<0.05) (Table 2), and the difference between the two groups became even more significant on day 52 (*P*<0.01). Body weight was also monitored to evaluate the general toxicities of these treatments. Figure 5C demonstrated that the body weight changes were within 20% in all groups, indicating no general toxicity in these treatments. Table 3 shows the mean tumor delay time and enhancement ratio of different treatments in the PC3 tumor-bearing model. The mean tumor growth delay time in the combination group was 42.2 days, and enhancement ratio caused by the combination treatment were 2.5 and 1.8 compared with castration alone and orlistat alone groups, respectively. As shown in Table 4, the CI in PC3 tumor-bearing mice was 0.651. This value was more than the expected inhibitory rate 0.576 which was obtained from [0.651 − (0.216 × 0.459) = 0.576], indicating that the combination of orlistat plus castration resulted in a synergistic effect. *Ex vivo* Western blotting (Figure 5D) showed that the expression of FASN, MMP-9, NF-κB p65, p-AKT, VEGF, p-ERK, cyclin D1 and Bcl-2 were all decreased; total t-AKT and t-ERK were not affected, while cleaved caspase-3 was increased in all three treatment groups. These results were similar to *in vitro* Western blotting findings. Notably, the combination treatment showed the most significant changes in protein expression levels. NF-κB activity was suppressed in all three treatment groups, with the most profound effect in the combination group as determined with the EMSA (Figure 5E). The original blot images of Figure 5D were shown in the Supplementary Figures S2 and S4.

**Table 2:** Tumor volume ratio statistical analysis

Days	Control vs. Castration	Control vs. Orlistat	Control vs. Combination	Castration vs. Orlistat	Castration vs. Combination	Orlistat vs. Combination
3	-	-	-	-	-	-
7	-	-	-	-	-	-
10	-	*	*	-	-	-
14	-	**	**	-	-	-
17	**	***	***	-	-	-
21	*	***	***	-	$	-
24	-	***	***	-	$$	-
28	*	***	***	-	$$	-
31	**	***	***	-	$$	-
35	-	***	***	-	$$$	-
38	*	***	***	$	$$$	-
42	*	***	***	$	$$$	-
45	-	***	***	$	$$$	-
49	*	***	***	$$	$$$	#
52	*	***	***	$$	$$$	##
56	*	***	***	$$	$$$	##

* *P*<0.05; ^**^P<0.01 and ^***^P<0.001, all treatments vs. control. ^$^*P*<0.05, ^$$^*P*<0.01 and ^$$$^*P*<0.001, orlistat and combination vs. castration. ^#^*P*<0.05 and ^##^*P*<0.01, orlistat vs. combination.

**Table 3:** Mean tumor growth time, delay time, inhibition rate and enhancement ratio in PC3 tumor-bearing mice after treatments with orlistat alone, castration alone and the combination of both

Treatment	MTGT (day)^1^	MTGDT (day)^2^	Mean growth inhibition rate^3^	Enhancement ratio^4^
Sham control	13.1	NA^6^	NA^6^	NA^6^
Castration (Cast)	22.5	9.40	1.72	2.45
Orlistat (ORL)	30.3	17.2	2.31	1.83
Cast+ORL^5^	55.3	42.2	4.22	-

The number of mice in each group was four to five, and all the experiments were repeated at least twice.

1 Mean tumor growth time (MTGT): the time at which the tumor volume ratio reached to 5.

2 Mean tumor growth delay time (MTGDT): the mean tumor growth time of the treated group minus that of the sham control group.

3 Mean growth inhibition rate: the mean tumor growth time of treated group/the mean tumor growth time of the sham control group.

4 Enhancement ratio: the mean growth inhibition rate of ORL+Cast group/the mean growth inhibition rate of ORL or Cast group.

5 ORL+Cast: combination treatment of orlistat plus castration.

6 NA: not available.

**Table 4:** Mean tumor growth inhibition rate and CI in PC3 tumor-bearing mice after treatments with orlistat alone, castration alone and the combination of both

Treatment	Mean growth inhibitory rate^1^	Expected growth inhibitory rate^2^	CI^3^
Sham control	-	-	-
Cast	0.216^*^	-	-
ORL	0.459^***^^$$^	-	-
Cast+ORL	0.724^***$$$##^	0.576	0.651 (synergism)

1 Mean growth inhibitory rate: 1 − (the 56^th^ day’s mean tumor volume ratio of treated group/the 56^th^ day’s mean tumor volume ratio of the sham control group).

2 Expected growth inhibitory rate: inhibition rates of ORL+Cast minus the multiplication of both ORL and Cast inhibition rates.

3 CI: (1 − mean growth inhibitory rate of Comb)/(1 − expected growth inhibitory rate).*P<0.05 and ***P<0.001, all treatments vs. sham control. ^$$^*P*<0.01 and ^$$$^*P*<0.001, orlistat and combination vs. castration. ^##^*P*<0.01, combination vs. orlistat.

### Pretreatment with orlistat combined with castration down-regulates the expression of FASN, p-AKT, p-ERK and NF-κB p65 in tumors from PC3 tumor-bearing mice

**Figure 6:**
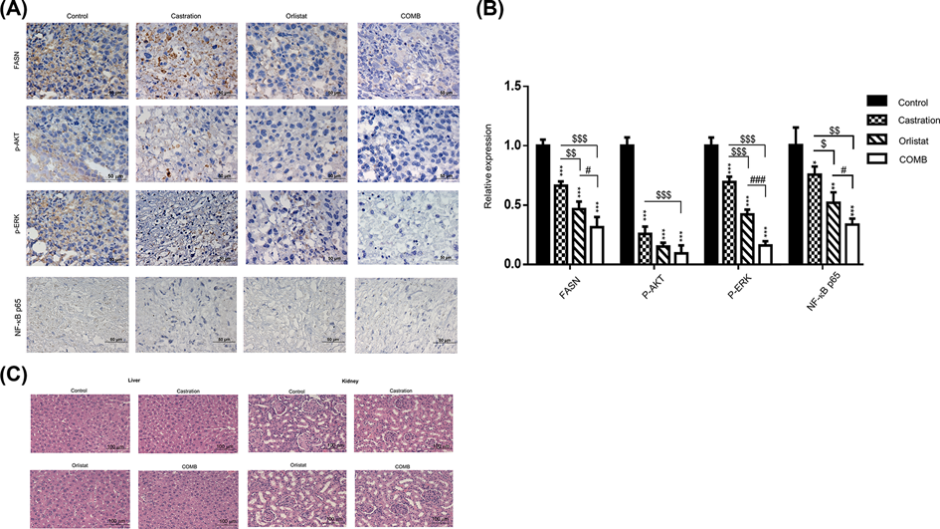
Combination of orlistat with castration synergistically inhibits the expression of FASN, NF-κB p65, p-AKT and p-ERK in PC3 tumor-bearing mice After 8 weeks of treatment, the mice were killed, and the tumors were collected. Immunohistochemistry was performed on tumor tissue samples for detection of FASN, p-AKT, p-ERK and NF-κB p65. (**A**) Blue and brown stains indicate the nucleus and the specific proteins, respectively. The results showed that the expression of FASN, p-AKT, p-ERK and NF-κB p65 were decreased by all three treatments with the most significant suppression in the combination treatment. Scale bar, 50 µm. (**B**) The expressions of FASN, p-AKT, p-ERK and NF-κB p65 in tumors as shown in (**A**) were quantified. (**C**) Histopathology of the liver and kidney. No tissue damage was found in both organs. Scale bar, 100 µm. *P<0.05, **P<0.01 and ***P<0.001, all treatments vs. control; ^$^*P*<0.05, ^$$^*P*<0.01 and ^$$$^*P*<0.001, orlistat and combination vs. castration; ^#^*P*<0.05 and ^###^*P*<0.001, combination vs. orlistat. All experiments were repeated at least three times. Abbreviation: p, phosphorylated.

Immunohistostaining was performed to further confirm the expression of several affected key proteins such as FASN, p-AKT, p-ERK and NF-κB p65 in tumors obtained from the animals (Figure 6A). The most significant suppression of these proteins were found in the combination group (Figure 6B). This finding was consistent with those of *ex vivo* Western blotting as shown in Figure 5D. Histopathological examination of the liver and kidney shown in Figure 6C demonstrated that no tissue or organ damage was found in the control and treatment groups.

### Biochemical analysis

Following 8 weeks of treatment, the mice were killed, and sera were collected for analysis of ALP, ALB, ALT, AST, BUN and CRE. The results were tabulated in Table 5.

**Table 5:** Biochemical analysis of sera obtained from mice killed on 56^th^ day post-treatments

Organ function	Control	Castration	Orlistat	Combination	Reference range
**Liver**					
ALP (U/l)	154 ± 29	149 ± 30	158 ± 37	144 ± 31	63–178
ALB (g/dl)	2.2 ± 0.2	2.6 ± 0.3	2.3 ± 0.3	2.3 ± 0.3	3.0–4.0
ALT (U/l)	127 ± 27	147 ± 47	170 ± 25	160 ± 50	27–78
AST (U/l)	48 ± 1	61 ± 21	104 ± 30	136 ± 28	20–215
**Kidney**					
BUN (mg/dl)	32 ± 6	28 ± 5	30 ± 8	33 ± 8	18–45
CRE (mg/dl)	0.3 ± 0.1	0.4 ± 0.2	0.2 ± 0.1	0.2 ± 0.1	0.3–0.5

Reference range of biochemical data was obtained from the Charles River Laboratories (Wilmington, MA, U.S.A.).

### MicroPET imaging

**Figure 7:**
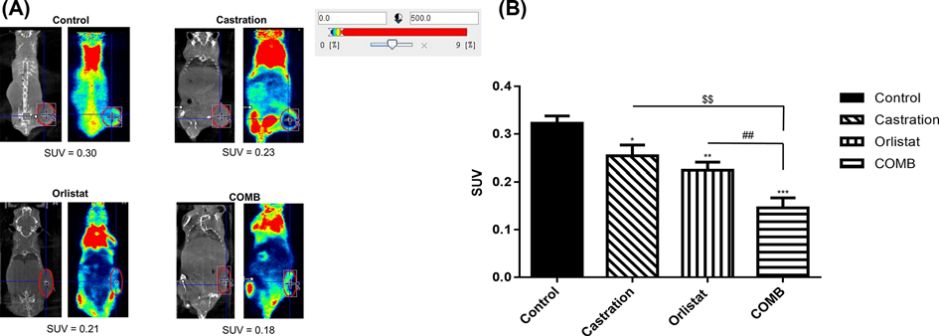
^18^F-FDG/microPET imaging was used for monitoring therapeutic response *in vivo* (**A**) On the 56^th^ day post treatment, microPET was performed at 30 min after intravenous injection of 19.6–20.1 MBq ^18^F-FDG, and images were acquired for 30 min. The experiments were repeated three times and one out of three is shown here. (**B**) The SUV of the tumors were quantified in different treatment groups (*n*=3/group). *P<0.05, **P<0.01 and ***P<0.001, all treatments vs. control. ^$$^*P*<0.01, orlistat and combination vs. castration. ^##^*P*<0.01, combination vs. orlistat. Experiments were repeated twice.

^18^F-FDG/microPET imaging could provide a non-invasive way to detect tumor progression and analyze the metabolic function of cancers. ROIs were drawn over the tumor area using the SUV. The SUVs of the tumors were 0.30, 0.23, 0.21 and 0.18 for the control, castration alone, orlistat alone and combination treatment, respectively (Figure 7A). The combination treatment showed the significantly lowest SUV among the treated groups (Figure 7B), which suggested the most effective tumor suppression.

## Discussion

FASN has been identified as an oncogene, is up-regulated in tumorigenesis, involved in the palmitoylation of Wnt1, cytoplasmic stabilization of β-catenin and activation of HER1/HER2 tyrosine kinase receptors [22,30,39,40]. In addition, FASN functionally plays a key role in *de novo* synthesis of long-chain fatty acids, which can be stored for metabolic energy and be constituted of lipids. Lipids, such as fatty acid and cholesterols, results in a more hypoxic and acidic tumor microenvironment, and lead to radioresistance in cancer [30]. Moreover, ionizing radiation could induce up-regulation of NF-κB and its upstream signaling molecules, such as AKT and ERK, and downstream effectors, such as E3 ubiquitin-protein ligase XIAP, Bcl-2 and MMP-9, resulting in radioresistance [41,42]. AKT inhibition has been shown to improve radiotherapy outcome [43,44], and a positive feedback loop between PI3K/AKT and FASN has also been proposed [45,46]. We have reported that inhibition of NF-κB using various drugs could enhance the therapeutic efficacy or improve radiotherapy outcome in PCa, oral cancer, hepatoma and colorectal cancer [37,47–52]. In addition, FASN plays an important role in tumor progression in AIPC, and is one of the causes behind CRPC development [24]. These indirect evidences suggested that FASN might be a potential target for the treatment of PCa.

The present study examined whether inhibition of FASN by orlistat could enhance the therapeutic efficacy of enzalutamide in the human androgen-independent PC3 prostate cancer cell line and ADT in tumor-bearing mice with multiple modules. The cytotoxicity was analyzed by AlamarBlue assay, and the results showed that orlistat could not only reduce the cell survival, but synergistically enhance the effects of enzalutamide on PC3 cells. Furthermore, the cell cycle distribution analysis showed that the G_0_/G_1_ proportion was significantly increased and the S-phase was significantly decreased upon orlistat treatment to PC3 cell cultures. These results are consistent with our previous study, which proved that inhibition of FASN by C75, another FASN inhibitor, could delay the progression of LNCaP cells [53]. As for the ADT, it was shown that ASJ-9 could suppress the proliferation and invasion of PCa cells during the castration-resistant stage by FASN inhibition [24]. In the current study, orlistat significantly enhanced the effects of enzalutamide on suppressing FASN, MMP-9, VEGF, cyclin D1 and Bcl-2 by inhibiting the phosphorylation of AKT and ERK. By restraining these proteins, the proliferation, invasion, migration, angiogenesis and anti-apoptosis abilities of PC3 cells were all limited.

AKT and ERK pathways are key activities in AIPC and radioresistance. Signal cross-talk between AKT and ERK could cause the growth of AIPC, the up-regulation of NF-κB and its downstream effector proteins, such as MMP-9, VEGF, cyclin D1 and Bcl-2, resulting in radioresistance [14,15]. NF-κB expression has been shown to increase the expression and activity of ARs in AIPC xenografts [54]. In our previous study, orlistat could improve radiotherapy outcomes in both LNCaP cells and PC3 cells via inhibiting FASN activity and phosphorylation of AKT pathway *in vitro* and reduce the tumor volume *in vivo* [37]. The present study demonstrated that orlistat synergistically enhanced the treatment outcomes of enzalutamide and ADT using castration in PC3 cells and tumor-bearing mouse model, respectively. The major signaling machineries involved the inhibition of both FASN and NF-κB activities via down-regulation of AKT and ERK pathways. This finding was in agreement with those reported by Wen et al. and Rae et al. [24,55]. Orlistat combined with castration in PC3 tumor-bearing mouse model showed the best therapeutic efficacy (Figure 5B). In our previous studies, we found that inhibition of FASN expression by orlistat could suppress tumor growth in both HT-29/*tk-luc* and LNCaP/*tk-luc* tumor-bearing mouse models [52,56]. In addition, Yoshii et al. used orlistat to treat LNCaP-, PC3- and DU145-bearing mouse models and found a significant reduction in tumor growth [57]. The body weight changes were within ±20% throughout the entire experiment. The e*x vivo* Western blotting and EMSA showed consistent findings as those results *in vitro*. The apoptosis induced by orlistat combined with castration was found via activation of cleaved-caspase 3. Overall, the results demonstrated that orlistat, castration and combination of both could down-regulate FASN expression, NF-κB activity and its effector proteins *in vivo*. Thus, these treatments may decrease the proliferation, invasion, migration and anti-apoptosis behaviors of cancer cells in PC3 tumor-bearing mice. Immunohistochemical (IHC) staining of FASN, p-AKT and p-ERK in tumors also confirmed the findings that these activated proteins were suppressed by orlistat alone, castration alone and the combination treatment, in which combination showed the most significant effect. In addition, no tissue damage was found in both the liver and kidney, as demonstrated by histopathology. Non-invasive ^18^F-FDG/microPET imaging further confirmed that combination of orlistat and castration showed the most effective tumor suppression compared with either treatment alone. Nevertheless, the diagnostic sensitivity of ^18^F-FDG/microPET imaging often is not satisfied due to its low uptake compared with that ^11^C-acetate in the PCa. Since the acetate metabolism is related to lipid synthesis, and FASN is an important enzyme during lipid synthesis. Therefore, ^11^C-acetate would be an ideal radiotracer to evaluate the FASN inhibition for the treatment efficacy in PCa. Orlistat combined with castration showed a common network involved the down-regulation of FASN, AKT, ERK, NF-κB and its downstream effector proteins. Moreover, the combination treatment exerted the best therapeutic efficacy and resulted in a synergistic effect compared with orlistat alone and castration alone, and suggested that FASN could be a potential target for clinical PCa therapy.

Aside from the androgen/androgen receptor signaling pathway, there are multiple intracellular signaling pathways involved in the signaling network and etiology of PCa. For instance, Wnt/β-catenin, NF-κB, JAK/STAT and RTK signal transduction pathways may also play a role in the development and progression of PCa, and essentially influencing the transition of PCa cells from androgen-dependent to androgen-independent and castration-resistant status [58]. From the molecular viewpoint, the NF-κB signaling pathway may not only affect the therapeutic outcomes of RT by inducing radioresistance, but also influencing the therapeutic efficacy of ADT in AIPC or CRPC [9,58]. The effects of FASN-targeted therapy by orlistat combined either with enzalutamide or castration could down-regulate the activity of NF-κB, which was closely associated with phosphorylations of AKT and ERK, resulting in tumor growth inhibition in AIPC. Orlistat combined with castration showed a common network involved the down-regulation of FASN, AKT, ERK, NF-κB and its downstream effector proteins. Moreover, the combination treatment exerted the best therapeutic efficacy with a synergistic effect compared with that of orlistat and castration alone, suggested that FASN could be a potential target for clinical PCa therapy. This is the first study, to our knowledge, demonstrates that FASN inhibition could sensitize androgen-independent PCa to enzalutamide *in vitro* and castration *in vivo*.

## Supplementary Material

Supplementary Figures S1-S4

## Data Availability

All supporting data are included in supplementary files. Raw data associated with the paper are available and can be accessed by contacting the authors.

## Abbreviations

ADT: androgen deprivation therapy
AIPC: androgen-independent PCa
ALB: albumin
ALP: alkaline phosphatase
ALT: alanine aminotransferase
AR: androgen receptor
AST: aspartate aminotransferase
BUN: blood urea nitrogen
CI: combination index
CRE: creatinine
CRPC: castration-resistant PCa
DAB: 3′,3′-diaminobenzidine
EMSA: electrophoretic mobility shift assay
FASN: fatty acid synthase
mHSPC: metastatic hormone-sensitive PCa
PBS-T: PBS with 0.05% Tween-20
PCa: prostate cancer
PI: propidium iodide
RT: radiation therapy
RTK: receptor tyrosine kinase
SUV: standardized uptake value
TBS-T: TBS-Tween-20
