# Supplementary material for: Effects of orlistat combined with enzalutamide and castration through inhibition of fatty acid synthase in a PC3 tumor-bearing mouse model
Source: Biosci Rep. 2021 May 27;41(5):BSR20204203. doi: 10.1042/BSR20204203 (PMC8164108; doi:10.1042/BSR20204203)
Supplement: Supplementary Figures S1-S4 [file BSR-2020-4203_supp.pdf]

Fig. S1

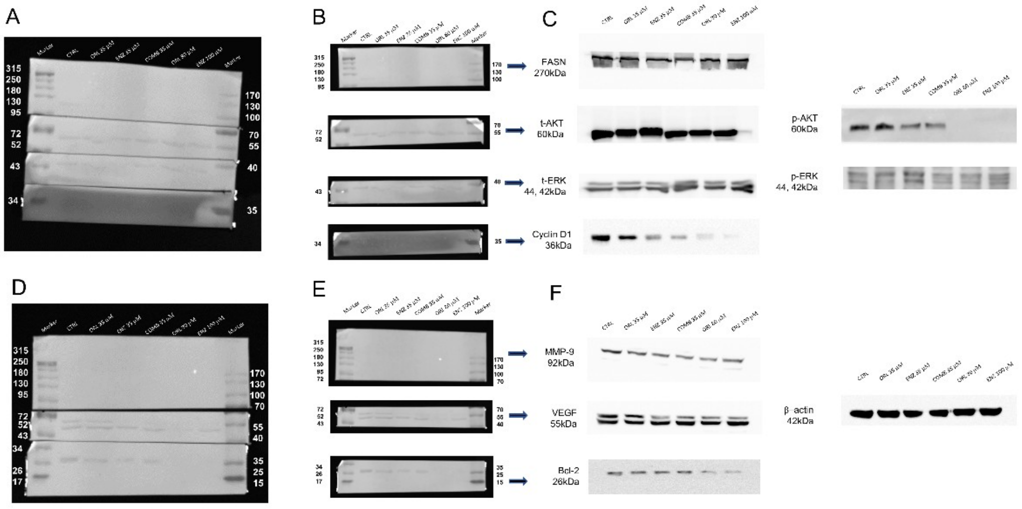

Figure S1. Original blot images presented in Figure 3.

Figure S1 shows the whole blot images after cutting membrane at molecular weight 95 kDa for FASN (270 kDa); 72 kDa for MMP-9 (92 kDa); 52 kDa for t-AKT (60 kDa) and p-AKT (60 kDa); 40 kDa for VEGF (55 kDa), t-ERK (44, 42 kDa), p-ERK (44, 42 kDa), ° β-actin (42 kDa); the remainder are Cyclin D1 (36 kDa) and Bcl-2 (26 kDa). (A) Original Western blot membrane (white light) for FASN, t-AKT, p-AKT, t-ERK, p-ERK, and Cyclin D1. This photo shows the cut membranes are put together to prove that these membranes are identical to those for Western blot. Since these photos were photographed under the white light, only markers could be seen. (B) Original Western blot membrane (white light). This photo shows the separated cut membranes. (C) Original Western blot membranes incubated with different antibodies and detected with chemiluminescence were shown with a one-to-one correlation to those of Figure S1 B. (D) Original Western blot membrane (white light) for MMP-9, VEGF, β-actin, and Bcl-2. Similar to (A), only markers could be seen. (E) Original Western blot membrane (white light). This photo shows the separated cutting membranes. Similar to (A), only markers could be seen. (F) Original Western blot membranes incubated with different antibodies and detected with chemiluminescence were shown with a one-to-one correlation to those of Figure S1 E.

Fig. S2

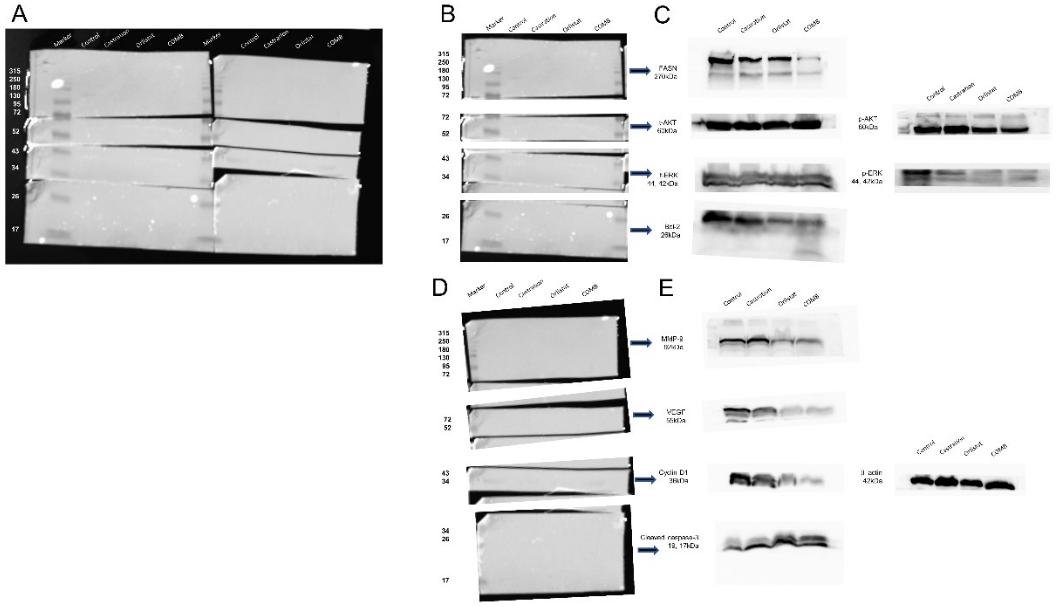

Figure S2. Original blot images presented in Figure 5D.

Figure S2 shows the whole blot images after cutting membrane at molecular weight 72 kDa for FASN (270 kDa) and MMP-9 (92 kDa); 52 kDa for t-AKT (60 kDa), p-AKT (60 kDa) and VEGF (55 kDa); 34 kDa for t-ERK (44, 42 kDa), p-ERK (44, 42 kDa), β-actin (42 kDa) and cyclin D1 (36 kDa); the remainder are Bcl-2 (26 kDa) and cleaved caspase-3 (19, 17 kDa). (A) Original Western blot membrane (white light) for FASN, t-AKT, p-AKT, t-ERK, p-ERK, and Cyclin D1. This photo shows the cut membranes are put together to prove that these membranes are identical to those for Western blot. Since these photos were photographed under the white light, only markers could be seen. (B) Original Western blot membrane (white light). This photo shows the separated cut membranes. (C) Original Western blot membranes incubated with different antibodies and detected with chemiluminescence were shown with a one-to-one correlation to those of Figure S2 B. (D) Original Western blot membrane (white light) for MMP-9, VEGF, β-actin, Bcl-2, and cleaved caspase-3. Similar to (A) only markers could be seen. (E) Original Western blot membranes incubated with different antibodies and detected with chemiluminescence were shown with a one-to-one correlation to those of Figure S2 D.

Fig. S3

A B

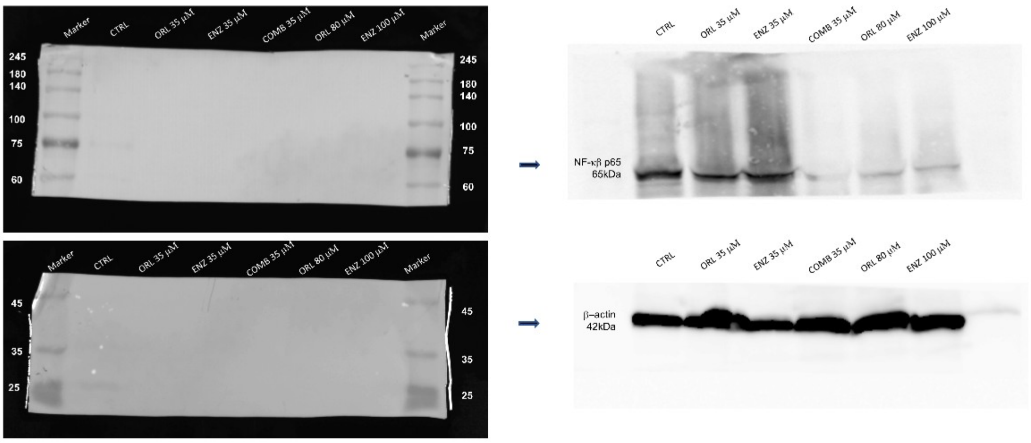

Figure S3. Original blot images of NF-κB p65 presented in Figure 3. (A) Original Western blotting membrane (light field). The upper panel shows the whole bolt images after cutting membrane at molecular weight 50 kDa for NF-κB p65 (65 kDa); and the lower panel is β-actin (42 kDa); (B) Blotting with different antibodies for original Western blotting membrane (one-to-one correlation).

Fig. S4

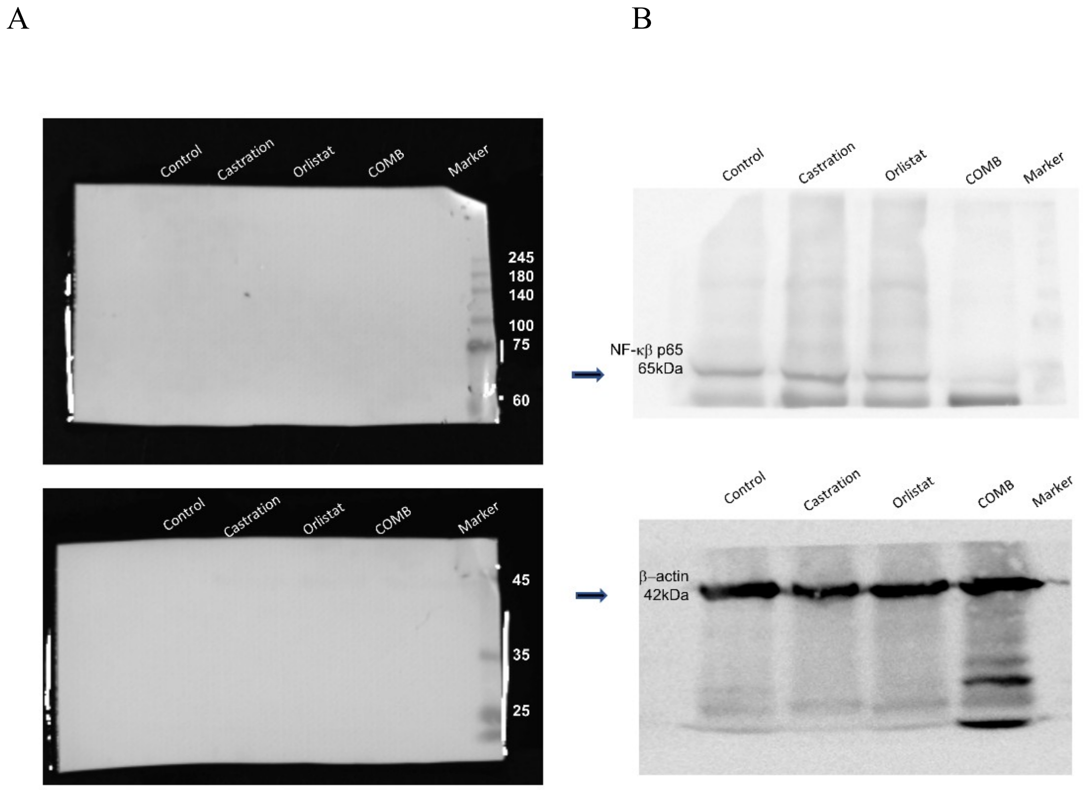

Figure S4. Original blot images of NF-κB p65 presented in Figure 5D. (A) Original Western blotting membrane (light field). The upper panel shows the whole bolt images after cutting membrane at molecular weight 50 kDa for NF-κB p65 (65 kDa); and the lower panel is β-actin (42 kDa); (B) Blotting with different antibodies for original Western blotting membrane (one-to-one correlation).
